# Giant Colonic Diverticulum: A Rare Type of Diverticular Disease

**DOI:** 10.7759/cureus.56463

**Published:** 2024-03-19

**Authors:** Jordan C Malone, Shiv R Patel, John P Walker, Marc Shabot

**Affiliations:** 1 Internal Medicine, University of Texas Medical Branch at Galveston, Galveston, USA; 2 John Sealy School of Medicine, University of Texas Medical Branch at Galveston, Galveston, USA; 3 Surgery, University of Texas Medical Branch at Galveston, Galveston, USA; 4 Gastroenterology and Hepatology, University of Texas Medical Branch at Galveston, Galveston, USA

**Keywords:** sigmoid colon, sigmoid colectomy, sigmoid diverticulitis, sigmoid diverticulosis, giant colonic diverticulum

## Abstract

Giant colonic diverticulum (GCD) is a well-recognized but infrequently encountered disease in clinical practice. GCD is its own unique entity and differs from commonly seen diverticular disease in both size and management. Initial clinical presentation is typically associated with diverticulitis and symptoms such as abdominal pain, fever, nausea, vomiting, rectal bleeding, or even a palpable abdominal mass. Surgery is the recommended treatment option largely due to the risk of associated complications including colonic perforation. We describe the case of a 56-year-old female diagnosed with a sigmoid GCD that was successfully stabilized medically and definitively treated surgically.

## Introduction

Protrusions of the colonic mucosa at weakened points of the muscular wall, known as diverticulosis, is a commonly encountered diagnosis in the general population [1]. However, giant colonic diverticulum (GCD) is a rare type of diverticular disease with many fewer cases reported in the literature. GCD is defined by any colonic diverticulum 4 cm or larger in diameter [2]. The management of GCD differs from typical diverticulosis. Surgery is the current first-line recommended treatment option given the high associated risk of complications including large bowel perforation. Herein, we describe the case of a 56-year-old woman who was diagnosed with this rare variant of diverticular disease after presenting with weeks of abdominal pain.

## Case presentation

A 56-year-old female with type 2 diabetes mellitus and hyperlipidemia presented to the emergency department in November 2023 with right lower quadrant and periumbilical abdominal pain of three weeks duration. She reported regular bowel movements and denied fever, nausea, vomiting, changes in bowel habits, or blood in the stool. Vital signs were normal. Physical exam showed tenderness to palpation below the umbilicus and in the right lower abdominal quadrant without rebound or guarding. No abdominal masses were palpable. She denied a family history of colon cancer and denied smoking and alcohol use. Her colonoscopy one year previous was endoscopically normal.

**Figure 1:**
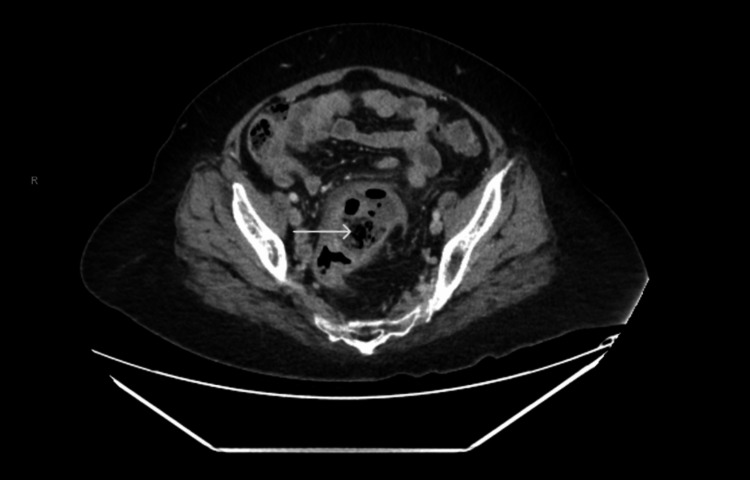
CT abdomen and pelvis (axial view) CT of the abdomen and pelvis (axial) demonstrating sigmoid GCD (arrow) with associated surrounding inflammation.

**Figure 2:**
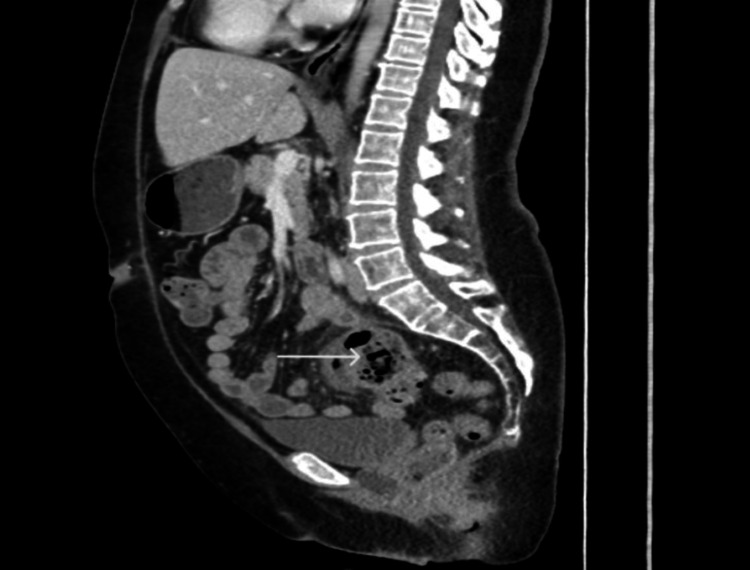
CT abdomen and pelvis sagittal view CT of the abdomen and pelvis (sagittal) demonstrating sigmoid GCD (arrow) with associated surrounding inflammation.

Lab work revealed a mild leukocytosis of 11,120 cells/µl. Computed tomography (CT) of the abdomen and pelvis with contrast revealed a large focal sigmoid diverticulum with associated inflammation and no evidence of perforation (Figures 1-2).

She was prescribed and completed a 10-day course of amoxicillin/clavulanic acid which initially improved her abdominal discomfort. She was evaluated by gastroenterology in the clinic roughly two weeks later with reports of initial improvement of her abdominal pain that had since relapsed following completion of her antibiotic course. She denied constipation, fever, or chills at this visit and was subsequently started on ciprofloxacin and metronidazole for an additional 14 days due to a recurrence of her abdominal pain while awaiting evaluation by colorectal surgery which was scheduled one week later. Colonoscopy was deferred at this time. 

**Figure 3:**
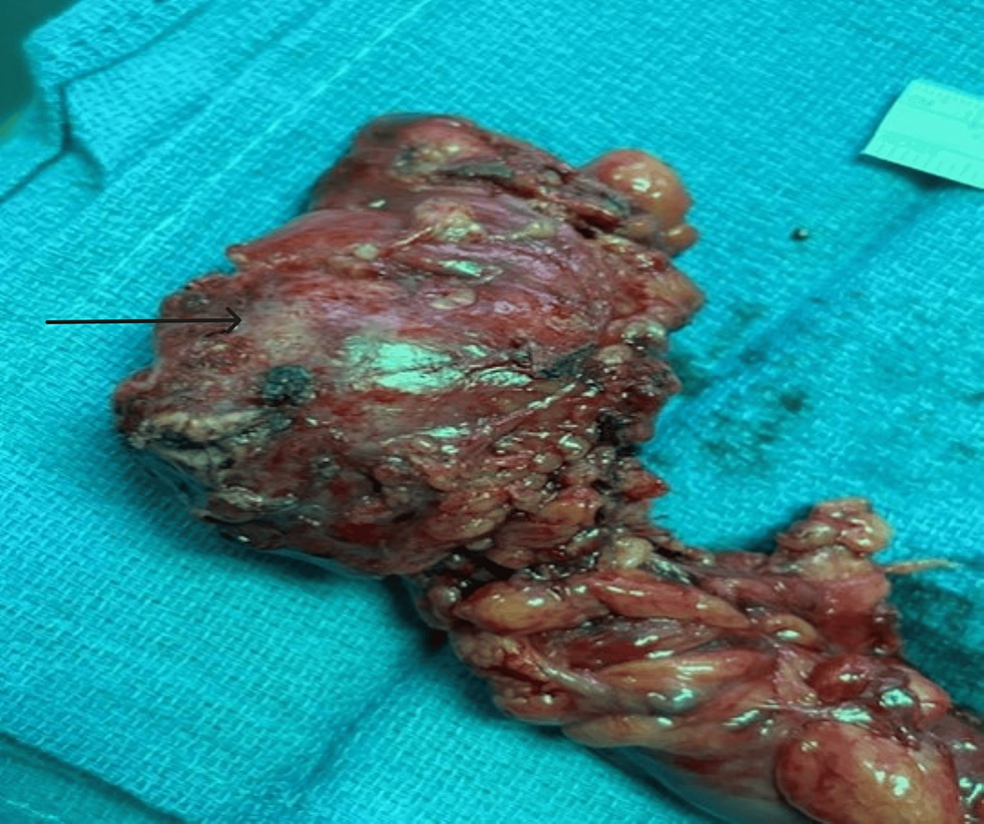
Resected sigmoid colon Gross surgical specimen demonstrating GCD (arrow) within resected sigmoid colon.

She was evaluated by colorectal surgery as mentioned and reported persistent abdominal pain ranked as 4/10 despite the extended antibiotic course. Plans were made for elective sigmoidectomy as the definitive treatment. She subsequently underwent laparoscopic sigmoidectomy one week later that revealed a firm and inflamed, 6 cm giant sigmoid diverticulum densely adherent to small bowel and small bowel mesentery that was sharply dissected and resected (Figure 3). 

The remainder of the procedure went without any complications. Pathological analysis revealed a 6 cm GCD with associated diverticulitis and surrounding granulation tissue formation, along with multiple other smaller sigmoid diverticula. Her perioperative course was unremarkable, and she reported feeling well at her most recent clinic follow-up visit.

## Discussion

This case highlights a unique type of diverticular disease and its preferred management. GCD is found in the sigmoid colon in 90% of cases but can be encountered anywhere throughout the large bowel [2]. Its pathogenesis is not fully understood, but some hypothesize that colonic air entrapment within the diverticulum or diverticular growth from gas-forming bacteria may play a role. They are occasionally found in isolation, but most are diagnosed with affiliated diverticulosis, such as in this case [2]. Manifestations of GCD can vary. Abdominal pain is the single most seen symptom and patients typically present with associated diverticulitis at the time of diagnosis.

The large size of GCD puts patients at higher risk for complications including perforation, obstruction, abscess and fistula formation, and bleeding. Perforation and abscess formation are the most common of these [3-4]. Complications occur in approximately 15-35% of cases and lead to mortality in about 5% of reported cases [4]. Associated underlying colonic malignancy has also been rarely documented [3].

Two classification systems specifically for GCD have been proposed. McNutt et al. divided GCD into three categorical subtypes: Type 1 diverticula are pseudodiverticula that lack a muscular layer [5]. Type 2 diverticula are thought to be due to inflammation and subserosal perforation, which creates a luminal communicating abscess cavity composed of fibrous tissue without the involvement of the colonic layers. Type 3 diverticula are true diverticula that contain all three layers of the bowel wall [5]. Choong et al. proposed another system with two distinct categorical types: Type 1 diverticula are pseudodiverticula that occur due to preexisting diverticular disease. Type 2 are true diverticula hypothesized to form secondary to communicating cystic congenital duplication [6].

CT enhanced with contrast is the preferred imaging modality to diagnose GCD. Typical appearance on CT shows a round, gas-filled structure often referred to as a “balloon sign” [7]. Colonic thickening of the suspected area is seen during the acute phase, and calcifications can also be present in patients with chronic disease [4]. Abdominal plain films can also detect GCD by showing evidence of a large, round, smooth-walled, radiolucent structure adjacent to the colon [2,5-6].

Definitive surgical treatment is recommended to prevent the complications previously outlined. Surgery involves resection of the affected colonic segment, which has shown good outcomes and long-term prognosis [2]. A minimally invasive approach, either robotic or laparoscopic, has demonstrated a quicker return to regular bowel function compared to an open approach [8]. Nonsurgical management is generally not recommended, but for patients deemed to be high risk or with contraindications to surgery, antibiotic therapy and percutaneous drainage of resultant abscesses have been employed [4].

## Conclusions

Although diverticular disease is common among adults, GCD is not. Clinicians need to recognize this uncommon type of diverticular disease to minimize unnecessary treatments and to expedite definitive surgical management to minimize the potential for complications. This case demonstrates an interprofessional approach to stabilization and subsequent definitive management of a large sigmoid GCD.

Prompt evaluation, diagnosis and treatment, and proper referral by the emergency room providers allowed expeditious follow-up with gastroenterology who provided further stabilization while awaiting definitive surgical management. This case highlights the importance of interprofessional communication to help facilitate proper workup and management of a rare diverticular entity, therefore minimizing the risk for the development of potentially life-threatening complications and optimizing patient outcomes.
